# Minimally invasive removal of deep contraceptive implants under continuous ultrasound guidance is effective, quick, and safe

**DOI:** 10.1007/s00330-021-08263-4

**Published:** 2021-10-14

**Authors:** Thibaut Jacques, Charlotte Brienne, Simon Henry, Hortense Baffet, Géraldine Giraudet, Xavier Demondion, Anne Cotten

**Affiliations:** 1 Division of Musculoskeletal Radiology, Lille University Hospital Center, Centre de Consultations Et D’imagerie de L’appareil Locomoteur, Rue du Professeur Emile Laine, 59037 Lille Cedex, France; 2 Lille University School of Medicine, Lille, France; 3 Division of Medical and Surgical Gynecology, Jeanne de Flandre Hospital, Lille University Hospital Center, Lille, France

**Keywords:** Contraception, Contraceptive devices, female, Radiology, interventional, Foreign bodies, Ultrasonography, interventional

## Abstract

**Objectives:**

The aim of this study was to assess the feasibility, performance, and complications of a non-surgical, minimally-invasive procedure of deep contraceptive implant removal under continuous ultrasound guidance.

**Methods:**

The ultrasound-guided procedure consisted of local anesthesia using lidocaine chlorhydrate 1% (10 mg/mL) with a 21-G needle, followed by hydrodissection using NaCl 0.9% (9 mg/mL) and implant extraction using a Hartmann grasping microforceps. The parameters studied were the implant localization, success and complication rates, pain throughout the intervention, volumes of lidocaïne and NaCl used, duration of the procedure, and size of the incision. Between November 2019 and January 2021, 45 patients were referred to the musculoskeletal radiology department for ultrasound-guided removal of a deep contraceptive implant and were all retrospectively included.

**Results:**

All implants were successfully removed en bloc (100%). The mean incision size was 2.7 ± 0.5 mm. The mean duration of the extraction procedure was 7.7 ± 6.3 min. There were no major complications (infection, nerve, or vessel damage). As a minor complication, 21 patients (46.7%) reported a benign superficial skin ecchymosis at the puncture site, spontaneously regressing in less than 1 week. The procedure was very well-tolerated, with low pain rating throughout (1.0 ± 1.5/10 during implant extraction).

**Conclusions:**

Minimally invasive removal of deep contraceptive implants under continuous ultrasound guidance alone is feasible, effective, and safe. In the present cohort, all implants were successfully removed, whatever the location, with short procedural time, small incision size, low pain levels, and no significant complications. This procedure could become a gold standard in this indication.

**Key Points:**

*• Minimally invasive removal of deep contraceptive implants under continuous ultrasound guidance alone is feasible, which led to a success rate of 100% whatever the location (even close to neurovascular structures), with only a small skin incision (2.7 ± 0.5 mm).*

*• The procedure was safe, quick, without any major complications, and very well tolerated in terms of pain.*

*• This minimally invasive ultrasound-guided procedure could become the future gold standard for the removal of deep contraceptive implants, as an alternative to surgical extraction, even for implants in difficult locations such as subfascial ones or those close to neurovascular structures.*

**Supplementary Information:**

The online version contains supplementary material available at 10.1007/s00330-021-08263-4.

## Introduction

In the USA and other western countries, hormonal implant is one of the main contraceptive methods, especially for 20–39-year-olds [1]. The percentage of women who have used it has more than doubled between 2002 (2.1%) and 2017 (5.6%) [2, 3]. Worldwide, it is used by about 23 million women [4]. It is one of the most effective means of contraception, with a Pearl index of 0.05 [5]**.** Nexplanon® is a flexible non-biodegradable 4 cm × 2 mm rod containing 68 mg etonogestrel and radio-opaque barium sulfate [6], with a duration of 3 years. It should be inserted subcutaneously on the medial side of the upper arm. However, in 0.25 to 1% of cases, insertion is too deep [7], especially in patients with low body mass index (BMI) [8], resulting in a greater risk of neurovascular damage due to local anatomy [9, 10]. There have also been cases of migration to pulmonary arteries via upper-limb veins [11–13].

When the implant is palpable and subcutaneous, the removal procedure is usually quick and performed without imaging guidance [5, 14–16]. On the other hand, non-palpable implants are liable to be too deep, e.g., under the brachial fascia or deep in the subcutaneous tissues, which makes their removal more complex. Indeed, nerve [17–20] or vessel [10] injuries have frequently been reported during the extraction in such cases. Therefore, patients with deep and/or non-palpable implants need to be taken care of in a referral center to minimize complications during the extraction procedure [8, 21].

When the implant is deep, ultrasound is the first-line means of localization, notably assessing supra- or sub-fascial position and the relationship to the surrounding neurovascular structures [22, 23]. The implant is easily located by its posterior acoustic shadow [24]. The most common procedure is for the radiologist to draw a skin landmark before the surgical extraction [21, 23, 25–27]. However, surgery involves soft-tissue dissection and a mean 15–20-mm incision for subfascial implants [23] and may still end up in failure in difficult cases [28].

Due to the high number of new contraceptive implants delivered each year, even a relatively low rate of nonpalpable cases (~ 1–3%) raises public health concerns. The aim of the present study was to assess, in a consecutive cohort of patients, the performance and complications of a novel minimally invasive procedure of deep hormonal contraceptive implants removal under continuous ultrasound guidance alone, under local anesthesia, using hydrodissection and microforceps [28].

## Methods

### Population

A single-center study was conducted in the musculoskeletal imaging department of the University Hospital of Lille, France. This is a referral center for the management of difficult implants, as described in the literature [8], with physicians (radiologists, gynecologists, surgeons) who have expertise in localizing and removing nonpalpable implants. Between November 26, 2019, and January 15, 2021, 45 patients were referred to the department specifically for the ultrasound-guided removal of a deep implant, defined as clinically non-palpable and/or with a failed attempt of removal in consultation or surgery. All consecutive 45 patients who underwent this intervention were included in this retrospective analysis. There was no loss to follow-up. Written informed consent was obtained for all patients for the analysis of the data from their procedure. Institutional Review Board approval was obtained under the reference CRM-2002–116.

### Procedure

Sterile equipment comprised a pair of gloves, an ultrasound probe cover, ultrasonography gel, compresses, antiseptic (povidone iodine), two syringes (10 cc and 20 cc), lidocaine chlorhydrate 1% (10 mg/mL), sodium chloride (NaCl 0.9%, 9 mg/mL), a scalpel, a 5-cm 21-G needle, wound closure strips, and 1 mm Hartmann grasping microforceps (MCO13A, Integra MicroFrance). The procedure was performed by a senior musculoskeletal radiologist experienced in ultrasound-guided interventions (T.J.). Procedures were performed on an outpatient basis in a dedicated interventional ultrasound room.

**Fig. 1:**
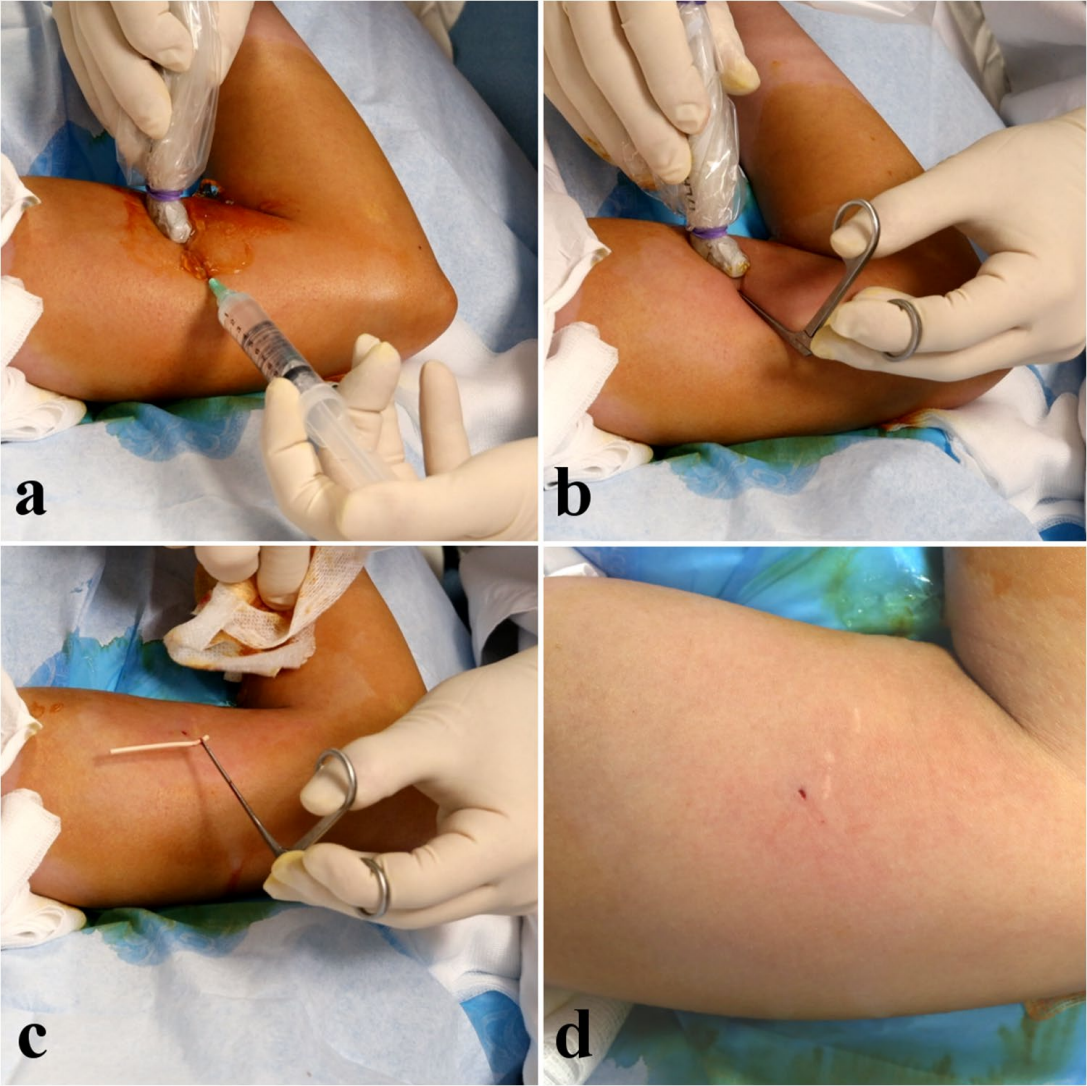
Successive steps of the procedure. Panel **a** shows the local anesthesia using a 21-G needle and lidocaïne 1%, followed by hydrodissection using NaCl 0.9% through the same needle. Panel **b** shows the insertion of the grasping microforceps through a skin incision made with the tip of a scalpel at the same insertion point. Panel **c** shows the en bloc removal of the implant. Panel **d** shows the final size of the incision, measuring 2 mm in this case, located near two previous scars (one from the insertion of the implant, the other from a previous failed attempt of clinical removal)

Patients were positioned supine, arm in 90–100° abduction, and external rotation, exposing the medial side. Ultrasound localization and removal procedures were performed using a high frequency hockey stick probe (i22LH8, Aplio i800, Canon Medical Systems). Under strict aseptic conditions and continuous ultrasound guidance, the radiologist implemented local anesthesia using lidocaine 1% (Fig. 1a) using a 5 cm 21-G needle, in all soft tissue up to the chosen grasping site of the implant. The needle was kept in the same position then used for hydrodissection by NaCl 0.9%, releasing adhesions around the grasping site of the implant and distancing local critical structures [29].

A small skin incision (tip of the scalpel) was made at the same insertion point to introduce the microforceps under ultrasound control (Fig. 1b). In contact with the implant, the forceps was opened to grasp it and remove it in a single piece, without fragmentation (Fig. 1c) (supplementary material 1).

The ultrasound probe was continuously kept parallel to the long axis of the forceps (“in-plane” forceps) and in the short axis of the implant (“out-of-plane” implant), which confers several advantages. First, it gives the operator a wider range of potential grasping sites along the 4 cm of the implant, whereas an “in-plane” approach of the implant would only enable a maximum of 2 grasping points (the implant edges). Moreover, due to its insertion technique, the implant runs parallel to the surrounding neurovascular structures in the medial side of the arm, a transverse approach thus enables a permanent control of both the implant and the surrounding structures. Finally, due to its flexibility and the soft nature of the surrounding tissues, the implant can move when in contact with the forceps, resulting in its disappearance out of the field of view if monitored in its long axis, which is not the case in its short axis since the implant is always on screen, even when pushed away or grasped by the forceps.

The size of the incision at the end of the procedure was measured (Fig. 1d) and skin closure was performed with wound closure strips.

Telephone follow-up at 1 week and 1 month screened for complications: local or general signs of infection, healing status, local or neuropathic pain, and local ecchymosis.

### Study data

Pre-procedural data comprised patient age, body-mass index (BMI: kg/m^2^), implant palpability, reasons for removal, and intended subsequent contraception in gynecological follow-up.

Data collected during ultrasound location comprised implant position (supra- or sub-fascial), implant depth with respect to the skin, and at-risk neurovascular structures within 3 mm of the implant (corresponding to the forceps opening distance).

Intra-procedural data comprised timing of the various steps (location, anesthesia, removal) and quantities of lidocaine 1% and NaCl 0.9% used. Pain was monitored throughout the procedure, on a 0–10 numeric scale.

### Statistics

Analyses were performed with Prism 9 software (GraphPad). Quantitative data were reported as mean ± standard deviation (SD). Normal distribution was tested using a D’Agostino-Pearson test. Normally distributed data were compared using the Welch t-test, and non-normal data using the Kolmogorov–Smirnov test. Qualitative data were reported as raw number and percentage (%) and compared using the two-tailed Fisher exact test. The significance threshold was set at *p* < 0.05.

## Results

### Patient and implant characteristics

Table 1 shows patient characteristics, and Table 2 shows implant characteristics.

**Table 1:** Characteristics of the population of patients

	Total (*n* = 45)		Suprafascial (*n* = 21)		Subfascial (*n* = 24)		
	Mean	SD	Mean	SD	Mean	SD	*p*
>Age (years)	30.5	8.2	31.3	9.9	29.7	6.5	0.51
BMI (kg/m^2^)	24.2	4.4	26.4	4.6	22.2	3.1	**0.001**
Main reason of removal Expiry date alone Hormonal symptoms Concern because of non-palpation Paresthesia Desire of pregnancy	***n*** 29 10 4 1 1	% 64.4% 22.2% 8.9% 2.2% 2.2%					
Subsequent contraception Contraceptive pill Intra-uterine device New contraceptive implant Vaginal ring None	**n** 20 7 7 1 10	**%** 44.4% 15.6% 15.6% 2.2% 22.2%					

*BMI* body mass index; *n* number of patients; *SD* standard deviation

Bold font was used to show statistically significant results

**Table 2:** Characteristics of the implants

	Total (*n* = 45)		Suprafascial (*n* = 21)		Subfascial (*n* = 24)		
	*n*	%	*n*	%	*n*	%	*p*
Non-palpable implant	41	91.1%	19	90.5%	22	91.7%	1.0
Previous removal attempt	11	24.4%	3	14.3%	8	33.3%	0.18
Neurovascular structure < 3 mm	19	42.2%	8	38.1%	11	45.8%	0.76
	**Mean**	**SD**	**Mean**	**SD**	**Mean**	**SD**	***p***
Subcutaneous depth (mm)	3.1	1.5	2.7	1.1	3.5	1.8	**0.04**

Bold font was used to show statistically significant results

**Fig. 2:**
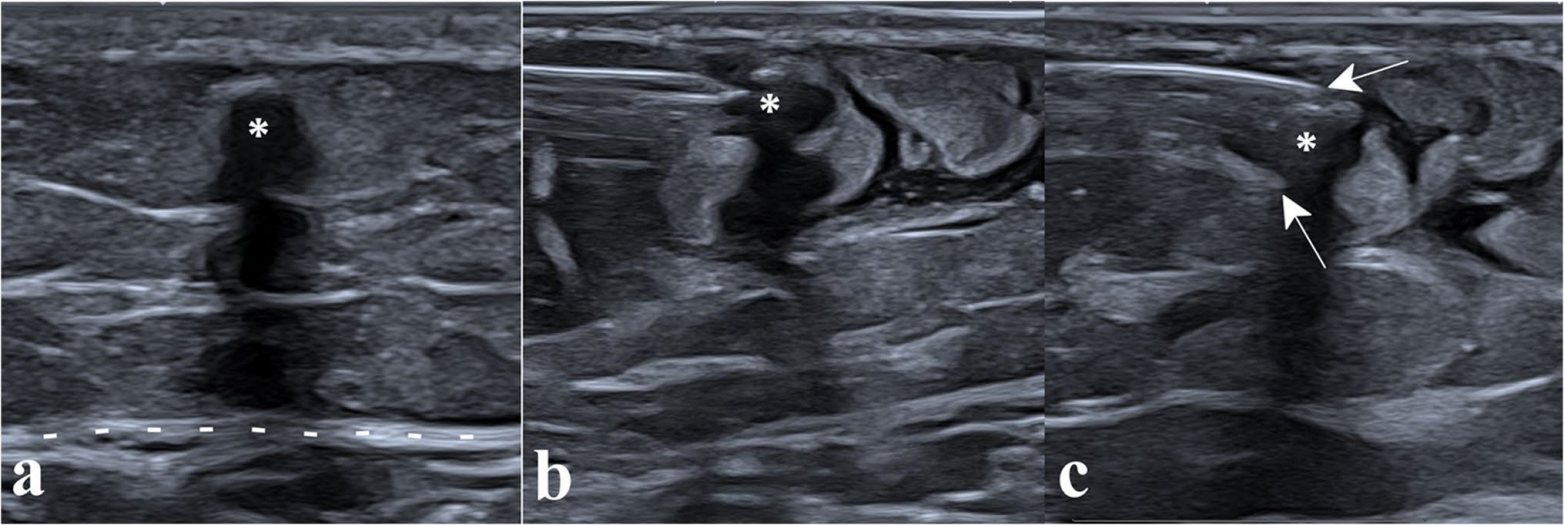
Removal of a suprafascial implant. Panel **a** shows the suprafascial implant (asterisk), seen in its short axis with posterior acoustic shadowing, located above the aponeurosis (dotted lines), in the subcutaneous soft tissues. Panel **b** shows the hydrodissection around the implant, using a 5-cm 21-G needle. Panel **c** shows the microforceps (arrows pointing at each jaw), introduced along the path of hydrodissection and open to grasp the implant

**Fig. 3:**
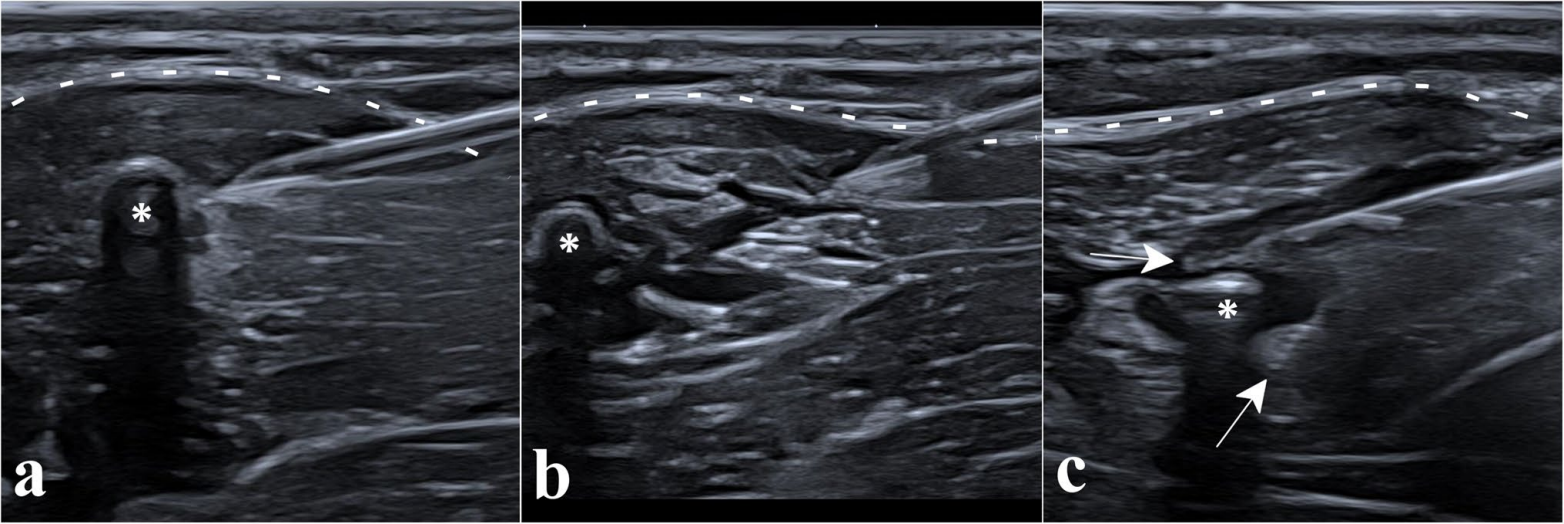
Removal of a subfascial implant. Panel **a** shows the subfascial implant (asterisk), seen in its short axis with posterior acoustic shadowing, located under the aponeurosis (dotted lines) inside the *biceps brachii* muscle; local anesthesia with lidocaine 1% was performed with a 5-cm 21-G needle from the superficial tissues to the implant. Panel **b** shows the hydrodissection of the surrounding tissues with NaCl 0.9% using the same needle, first around the implant (asterisk) then during the removal of the needle to prepare the path for the microforceps. Panel **c** shows the open microforceps (arrows pointing at each jaw) introduced along the same path to grasp the implant (asterisk)

Twenty-one of the 45 implants (46.7%) were suprafascial (Fig. 2), and 24 (53.3%) were subfascial (Fig. 3); 41 implants (91.1%) were non-palpable the day of the procedure.

The mean age was 30.5 ± 8.2 years. Mean BMI was 24.2 ± 4.4 kg/m^2^, and significantly lower in subfascial cases: 22.2 kg/m^2^ versus 26.4 kg/m^2^ for suprafascial cases (*p* = 0.001).

**Fig. 4:**
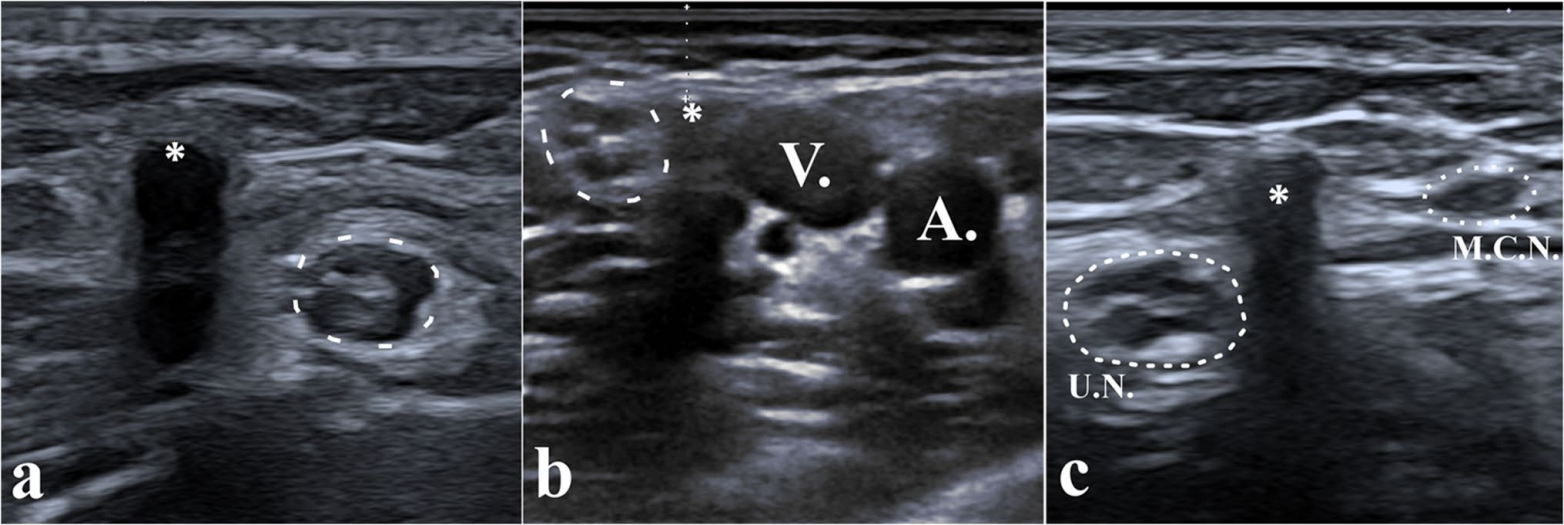
Implants located in close proximity to neurovascular structures, more at risk of damage during removal. Panel **a** shows a suprafascial implant (asterisk) located only 1.8 mm away from the ulnar nerve (dotted line). Panel **b** shows a subfascial implant (asterisk) located only 3 mm subcutaneously (calipers) but between the ulnar nerve (dotted line) and the basilic vein (V), and close to the brachial artery (A). Panel **c** shows an implant (asterisk) located < 2 mm to both the ulnar nerve (UN) on one side and the Medial Cutaneous Nerve of the arm (MCN) on the other side. These three implants were removed safely without complication using the reported technique

Mean implant depth below skin was 3.1 ± 1.5 mm, subfascial implants being significantly deeper to the skin surface than suprafascial implants (3.5 ± 1.8 mm versus 2.7 ± 1.1 mm respectively, *p* = 0.04). Nineteen implants (42.2%) were located within 3 mm of a neurovascular structure (Fig. 4), without significant difference according to supra- or sub-fascial location (8 supra- versus 11 sub-fascial; *p* = 0.76).

Eleven patients (24.4%) had experienced 1 or 2 previous failed attempts of removal: surgically (*n* = 3) or clinically (*n* = 8 office procedures by GP, gynecologist, or midwife), without significant difference depending on the location of the implant (*p* = 0.18).

### Results of procedure

All implants were successfully removed en bloc (100%). The mean incision size, measured at the end of the procedure, was 2.7 ± 0.5 mm.

Mean total procedure time (location of the implant using ultrasound, equipment preparation, skin asepsis, anesthesia then extraction) was 19.2 ± 9.0 min, significantly longer for subfascial implants compared to suprafascial ones: 22.9 ± 9.0 versus 15.0 ± 7.1 min respectively (*p* = 0.004).

The mean extraction time as such (from the onset of local anesthesia to implant removal) was 7.7 ± 6.3 min, significantly longer for subfascial implants: 10.0 ± 6.7 versus 5.0 ± 4.7 min for suprafascial ones (*p* = 0.01).

The mean saline volume used for hydrodissection was 5.7 ± 4.4 mL and significantly greater for subfascial implants: 8.7 ± 3.7 versus 2.3 ± 2.3 mL for suprafascial ones (*p* < 0.0001). The mean volume of lidocaine 1% used for local anesthesia was 4.4 ± 1.9 mL, which was also significantly higher for subfascial implants compared to suprafascial ones: 4.9 ± 1.9 mL versus 3.9 ± 1.8 mL respectively (*p* = 0.02).

In terms of pain, the procedure was very well-tolerated, with low pain rating throughout the intervention: 0.6 ± 1.0/10 at the onset of anesthesia, 1.0 ± 1.5/10 during implant extraction, and 0.2 ± 0.8/10 at the end of procedure.

### Post-procedural data

In the immediate post-procedural phase, 1 patient (2.2%) showed vagal prodroma and 2 (4.4%) showed transient paresthesia in the territory of the medial cutaneous nerve of the arm, spontaneously regressive before the end of the procedure, without further symptoms.

There were no infections, hemorrhagic complications, or cases of neuropathic pain at day 7, only minor complications: 21 patients (46.7%) reported a superficial skin ecchymosis at the puncture site, spontaneously regressive within the first week, not requiring special care nor complementary examinations, and without significant difference between groups (45.9% in the subfascial group versus 47.7% in the suprafascial group) (*p* > 0.99).

## Discussion

The aim of the present study was to assess, on a consecutive cohort of patients, the feasibility, effectiveness, and safety of minimally invasive removal of deep contraceptive implants under continuous ultrasound guidance, as previously described in one case [28]. The use of this technique enabled both localization and removal of deep contraceptive implants in the same procedure, without resorting to open surgery. It may optimize precision, is feasible under local anesthesia on an outpatient basis, and appears less invasive than a surgical approach.

The success rate in our cohort of 45 consecutive patients was 100%, including for locations considered as difficult in the literature: subfascial and/or close to neurovascular structures [8, 21]. Continuous ultrasound guidance is a key point and enabled a safe procedure thanks to a permanent control of the surrounding soft tissues. Hydrodissection was useful to create safe areas between the implant and surrounding structures.

Removal time varied significantly according to location: subfascial implants required stronger hydrodissection at the fascia and then crossing the fascia with the forceps, accounting for the slightly longer procedure and greater quantity of saline. The rate of neurovascular structures within 3 mm of the implant did not significantly differ according to sub- or supra-fascial location. Some subfascial implants, especially when intramuscular, were remote from any neurovascular structure, while some suprafascial ones were separated by only a thin fascia or were close to a suprafascial nervous branch (such as medial cutaneous branches), highlighting the need for precise prior ultrasound localization.

There were no major complications such as infection or vascular or neural injury, regardless of implant location. The main minor complication was superficial ecchymosis at the puncture site in 46.7% of cases, requiring neither analgesia nor complementary examination, spontaneously regressing in less than 1 week. In terms of pain, the procedure was very well tolerated, with pain levels remaining very low throughout the intervention. As in the study by Matulich et al., the present results confirm that low BMI is significantly associated with subfascial implant location [8].

Finally, this minimally invasive technique allowed a less traumatic approach than with surgical dissection, using a single small incision averaging 2.7 mm, whatever the implant location, as opposed to a mean of 15–20 mm for surgical removal of subfascial implants according to recent reports [23], thus leaving a smaller scar. The esthetic result is important, considering the visible location of the site (on the upper arm), in patients who are young.

The concept of ultrasound-guided foreign bodies removal is not new to the radiology literature [30]. Regarding the specific indication of contraceptive implants, few series have been published, yet none using real-time ultrasound control during the whole procedure, the technique we describe here has thus not been (to our knowledge) previously described. The most common use of ultrasound in the literature has been for the localization of the implant, skin-to-depth measurement, and skin landmark, such as in the recent publication by Kim et al.[31], but the removal was performed using open surgical dissection, without real-time imaging guidance during the removal procedure itself. An older publication by Nelson and Sinow [32] used ultrasound to locate and stabilize the implant with a needle, but required an open surgical incision of the fascia in the cases of subfascial or intramuscular implants, which was not required in the procedure we report here. The same kind of intervention was described by Persaud et al.[33], where ultrasound played a key role mainly for the localization of the implant, skin marking, and intermittent control during the procedure, but the extraction itself was performed using a blunt open soft tissue dissection and skin holding forceps. Publications by Guiahi et al.[34] and Chen et al.[35] both used a vasectomy clamp after implant localization by ultrasound; the direct puncture with the clamp through an incision made over the implant location did not enable a continuous ultrasound monitoring of the implant and soft tissues during removal. The control during the removal procedure was either clinical or under fluoroscopic guidance, but none enabled a real-time ultrasound control of adjacent soft tissues, as opposed to the procedure we describe here.

The main study limitation was the single-center design: all procedures were performed by the same radiologist specialized in musculoskeletal imaging, with experience in ultrasound-guided procedures. Wider use of the procedure would require practical training for users performing ultrasound-guided procedures. There could however be training issues for non-specialist personnel in replicating the technique. The results of the current study thus need to be extrapolated with caution in different clinical settings.

## Conclusion

Minimally invasive removal of deep contraceptive implants under continuous ultrasound guidance is feasible, effective, and safe. In the present cohort, all implants were successfully removed, whatever the location, with short procedural time, small incision size, low pain levels, and no significant complications. This procedure could become a gold standard in this indication.

## Supplementary Information

Below is the link to the electronic supplementary material.

Supplementary file1 (MP4 81784 KB)

## Abbreviations

BMI: Body mass index
G: Gauge
NaCl: Sodium chloride
SD: Standard deviation

## Declarations

### Guarantor

The scientific guarantor of this publication is Dr. Thibaut Jacques.

### Statistics and biometry

No complex statistical methods were necessary for this paper.

### Methodology

• retrospective

• observational

• performed at one institution
